# Immunization against *Pseudomonas aeruginosa* using Alg-PLGA nano-vaccine

**DOI:** 10.22038/ijbms.2021.52217.11813

**Published:** 2021-04

**Authors:** Saeid Azimi, Leila Safari Zanjani

**Affiliations:** 1 Parseh Institute of Iran, Tehran, Iran; 2 Department of Cellular and Molecular Biology, Zanjan Branch, Payame Noor of Zanjan, Zanjan, Iran

**Keywords:** Alginate, Cytokine, Opsonophagocytosis, PLGA, Pseudomonas, aeruginosa

## Abstract

**Objective(s):**

*Pseudomonas aeruginosa* is the bacterium that causes of pulmonary infection among chronically hospitalized patients. Alginate is a common surface antigen of *P. aeruginosa* with a constant structure that which makes it an appropriate target for vaccines. In this study, *P. aeruginosa *alginate was conjugated with to PLGA nanoparticles, and its immunogenicity was characterized as a vaccine.

**Materials and Methods:**

Alginate was isolated from a mucoid strain of *P. aeruginosa* and conjugated with to PLGA with˝ N-(3-Dimethylaminopropyl)-N′-ethylcarbodiimide hydrochloride ˝= ˝EDAC˝ and N-Hydroxysuccinimide (NHS). Chemical characterization of prepared nano-vaccine was performed using FTIR Spectroscopy, Zetasizer, and Atomic Force Microscopy (AFM). The immunogenicity of this nano-vaccine was evaluated through intramuscular injection into BALB/c mice. Four groups of mice were subjected to the injection of alginate–PLGA, and two weeks after the last administration step, opsonophagocytosis assay, IgG detection, challenge, and cytokine determination via ELISA were carried out.

**Results:**

Alginate-PLGA conjugation was corroborated by FTIR, Zetasizer, and AFM. The ELISA consequence showed that alginate was prospering in the instigation of the humoral immunity.The immunogenicity enhanced against the alginate-PLGA. Remarkably diminished bacterial titer in the spleen of the immunized mice posterior to challenge with PAO1 strain in comparison with the alginate alone and control groups.

**Conclusion:**

The bacterial burden in the spleen significantly decreased after the challenge (*P*<0.05). The opsonic activity was significantly increased in the alginate- PLGA group (*P*<0.05).

## Introduction

The mucoid *Pseudomonas aeruginosa* is among the most important causes of pulmonary infection in Cystic Fibrosis (CF) patients. This patient correlates with alginate-producing *P. aeruginosa*. The bronchial obstruction due to viscous mucus secretion and poor inhibition of colonization of mucoid *P. aeruginosa* results in a high incidence of pulmonary infection (1-4).

*P. aeruginosa *strains often have mucoid colony morphologies, due to the over production of the alginate in this bacterium. Alginate, which is a linear copolymer of partially O-acetylated b -1, 4-linked D-mannuronic acid and L-guluronic acid is the main component of the *P. aeruginosa *biofilm matrix. Alginate, that is also called Mucoid Exopolysaccharide(MEP), is produced by mucoid strains of *P. aeruginosa *and significantly increases the resistance of these organisms to antibiotics treatment and host defenses and decreases the chemotaxis of polymorphonuclear cells. It prevents the complement activation and suppresses phagocytosis by neutrophils and macrophages, especially in CF patients (5-11).

Alginate is not solely expressed in mucoid strains. Indeed, its synthesis is also increased in non-mucoid strains of *P. aeruginosa* exposed to a hypoxic milieu. This phenomenon is observed in the lungs of CF patients within the mucus plugs in the airway (12).

As alginate has very slight structural variations, is considered as an appropriate target for developing new vaccines. There are different alginate vaccines for *P. aeruginosa*. To enhance its immunogenicity, alginate has been conjugated to carrier proteins (for instance, binding thiolated MEP (mucoid exopolysaccharide), to keyhole limpet hemocyanin (KLH), its covalently coupling to *P. aeruginosa* toxin A or alginate–tetanus toxoid (TT) conjugate, etc(13-18, 20, 22, 26,38, 46). 

Polymeric nanoparticles (NPs)based on glycolic acid (nanoparticles) are widely used in the reined escape of antigen, because of their advantages like biodegradability, biocompatibility, low toxicity, and site-specific delivery (26-29) .

In the present study, we built and characterized candidate vaccines based on *P. aeruginosa* alginate using PLGA nanoparticles. Then the efficacy of the Alg-nanoparticles that is stimulating the immune responses was assessed *in vivo* and *in vitro*.

## Materials and Methods

***Bacterial strains***

In this research, alginate was produced using two standard strains of *P. aeruginosa*, i.e. PAO1, and mucoid strain 8821M (Kindly provided by Dr Sobhan Faezi, Medical Biotechnology Research Center, Paramedicine Faculty, Langarud, Iran). It is necessary to mention that, the strain 8821M was used only for the extraction of alginate and we have used the PAO1 strain for the other tests such as challenge, opsonophagocytic, the titer of antibody and cytokine examination.

***Extraction and purification of alginate***

Alginate was purified according to the protocol proposed by Htatno *et al.* with some modifications. The PAO1 strain of *P. aeruginosa* strain (ATCC15442) was inoculated into the synthetic medium (pH of 7.5) including 10.1 ml/l glycerol, 0.5 g/l glucose, 0.37 g/l L-glutamine, 0.6 g/l NaHPO_4_, 0.12 g/l K_2_HPO_4, _ and 0.13 g/l MgSo_4_.7H_2_O (all materials were bought from Merck, Germany) at 37 °C for three days. The culture was inactivated by adding 4.5 ml of 90% savlon and incubated at 60 °C for 15 min, followed by repeated centrifugations (at 18000 × g and 4°C for 30 min) to pellet the bacterial cells. Thereafter, the resulting supernatant containing alginate was collected, filtered, and incubated at 4 °C for at least 8 hr.

 After adding ice-cold ethanol (Merck, Germany) with a volume three-folds greater than that of supernatant and subsequent incubation at 4 °C overnight, the precipitated alginate was collected through centrifugation (3500 × g at 4 °C for 15 min) and dissolved in Tris buffer (pH 8.0) containing 5% SDS (Merck, Germany), 10 mM CaCl_2_ (Merck, Germany) and proteinase K (10 μg/ml, Bioneer, South Korea) and incubated at 56 °C for 2 hr. To remove any remaining DNA and RNA contamination, DNase I and RNase A (at 100 mg/ml concentrations, Bioneer, South Korea) were used.

 A mixture of the sample : phenol : chloroform (2:1:1) was added and then incubated at 60 °C for 45 min. After centrifugation (40000 × g at 22 °C for 20 min), the supernatant was collected and mixed with an equal volume of chloroform.

 After 8 min of incubation, the tube was centrifuged (40000 × g at 22 °C for 40 min) and then dialyzed against dH2O for three days and finally lyophilized. For alginate isolation, the sample was applied to a XK 16 column (2.6 × 100 cm) packed with a Sephacryl S-400 gel filtration column (GE Healthcare, Life Sciences, Swaziland). The eluted tubes were evaluated for the uronic acid content at 595 nm(23-25).

***Conjugation of alginate into PLGA***

Conjugation of alginate into PLGA was done according to the protocol proposed by Safari Zanjani and Azimi (61). With minor modified, by replacing the alginate antigen at the end of the protocol. Then, chemical characterization of the conjugated alginate-PLGA was carried out (19, 21).

***Analytical methods for alginate-PLGA characterization***

The conjugated alginate PLGA was characterized using a (FTIR) spectrophotometer (Bruker, Germany), (AFM) (NanoWizard, Germany), and Zetasizer (Nano ZS, England) (43). 

***Immunization of mice***

Mice (BALB/c, weighing 20 to 25 g, 6-8 weeks old) in four groups (Alg -PLGA, Alginate, PLGA, and PBS (control group)) were intramuscularly immunized. Each group included six mice (three mice for bacterial enumeration in the spleen and three for sera isolation and opsonic killing activity, antibody titers, and cytokines response). The mice in each group were immunized using 10 μg (in accordance with the standard) of their corresponding antigen.

 These mice were immunized three times with two weeks, interval. Prior to the first immunization and two weeks after each immunization, blood samples were collected and sera were isolated through centrifugation (41, 42, 51, 54). 

***Enzyme-linked immunosorbent assay***

Detection of antibody titer to the nanovaccine was performed through ELISA assay by Safari Zanjani and Azimi (61, 62). 

***Challenge test***

*Pseudomonas aeruginosa* inoculum was incubated on BHI broth from a fresh overnight culture of *P. aeruginosa* PAO1 on BHI agar, under agitation (180 rpm) at 37 °C for 3-4 hr. The cells (OD_620_ nm = 0.18) were centrifuged and re-suspended in a sterile BHI broth. 

The plating serial dilutions were used to determine the number of bacteria. Fourteen days after the last immunization, all mice (immunized with Alg-PLGA, Alg alone, PLGA, and control groups) were challenged with the PAO1 strain of *P. aeruginosa* (1.5 × 10^8^ CFUs) through the peritoneal injection route. 

After the 72 hr from the challenge, the mice were killed and their spleens were harvested and homogenized in 10 ml of PBS (pH 7.4). Finally, serial dilutions of homogenates were plated onto Nutrient agar in triplicate and CFUs were calculated after two days of incubation at 37 °C (32, 47-50).

***Opsonophagocytosis test***

Opsonophagocytosis test was performed according to the method described by Safari Zanjani and Azimi (61, 62).

***Cytokine test***

Mice were intramuscularly injection three times, with fourteen days intervals. 14 hr after the last injection, blood samples were taken, centrifuged (10000g, 15 min) and frozen before performing the cytokine test. The cytokines, i.e. TNF-a, IL-4, IL-17A, and INF-γ were quantified through Enzyme-linked immunosorbent test. Cytokine test were done using different kits (all from Mabtech Ebioscience R&D, USA) according to the manufacturer’s instructions.

This test was described by Safari Zanjani and Azimi. with minor modified (31, 32,62).

***Statistical review***

For statistical review, the Graph-Pad Software version 6.0 for Windows, (San Diego, CA, USA) was used. Data were done using Tukeys test (ANOVA). Kaplan-Meier survival curves and the log-rank test were used to analyze different groups. All data were expressed as mean±SD and *P-* values less than 0.05 were considered to be significant.

**Figure 1:**
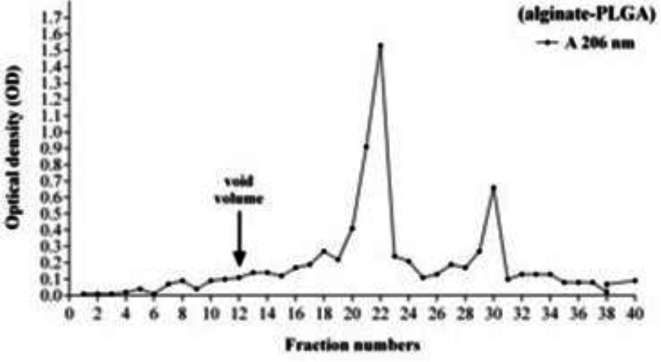
The chromatography of purified alginate-nanoparticles using Sephacryl S-200 HR. Three ml tubes were floced and analyzed due to the optical density (OD)

**Figure 2:**
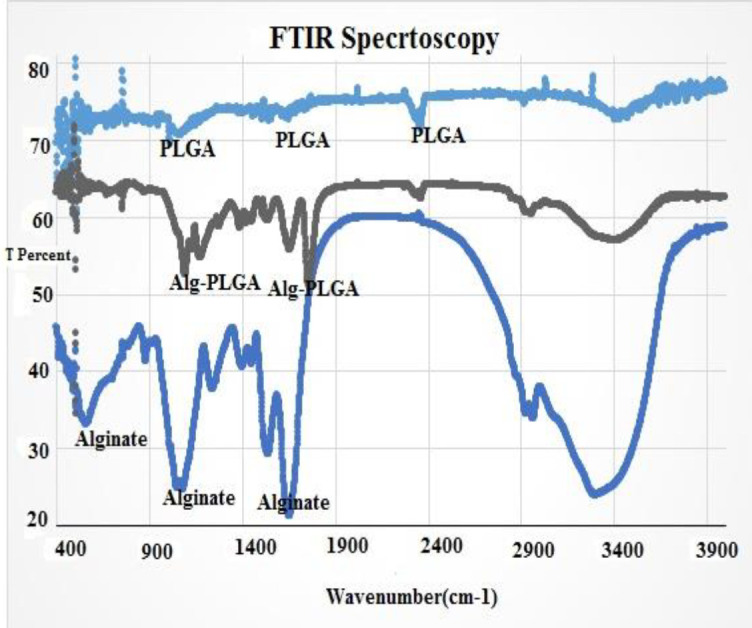
FTIR test of nanoparticles, Alginate, and Alginate-nanoparticles conjugates

**Figure 3:**
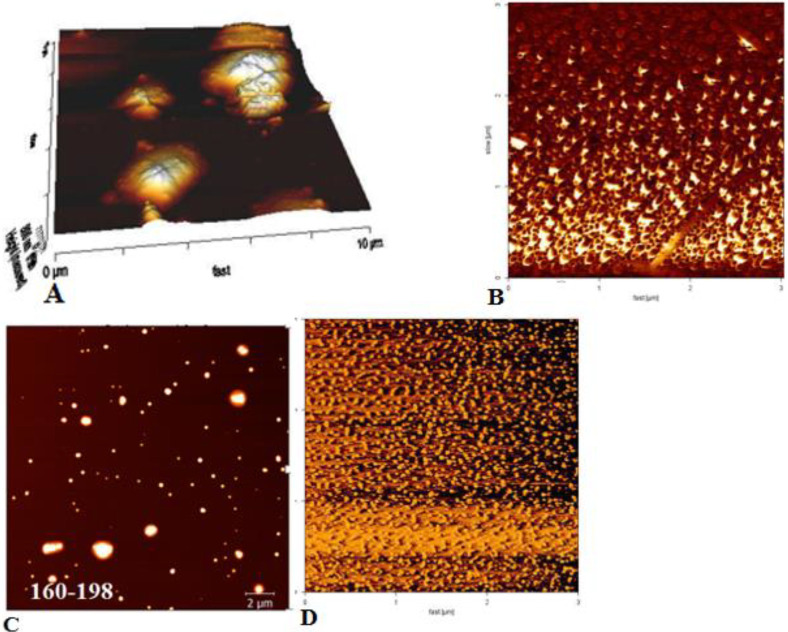
Atomic Force Microscopy (AFM) pictures of conjugated alginate in nanoparticles. (A) 2-D mapping and B) 3-D mapping of nanoparticles afore the conjugation of alginate. After conjugation, the size of PLGA was enhanced in alginate-nanoparticles (Cand D)

**Figure 4:**
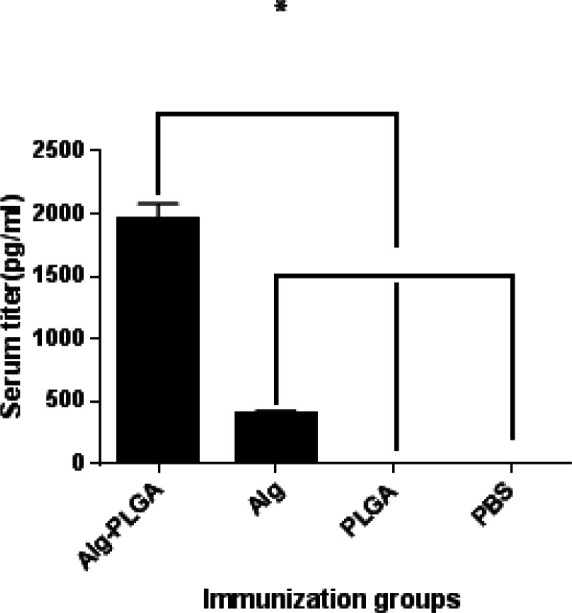
Evaluating the antibody titer to alginate in the immunized mice. Alg-specific IgG consequences are the mean of three mice in eperimental groups. Bars represent mean±SD from three mice per group. ND demonstrates the non-scrutable difference and * demonstrates to be significant at (*P*<0.05) among Alg-nanoparticles with other groups. Moreover, there was no significant difference at (*P* < 0.05) among nanoparticles alone with the control group (PBS)

**Figure 5:**
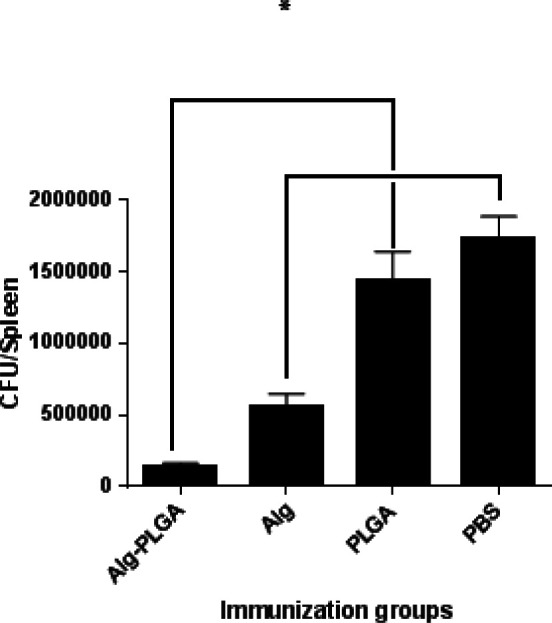
Challenge test. 72 hr since infection with PAO1 strain of *Peudomonas aeruginosa*, the spleen of mice was separated and homogenized. The serial diluted of homogenates were plated for P.aeruginosa numeration. Bars represent the means of duplicate determinations, and error bars indicate SD. Consequences were admitted to be significant at (*P*<0.05). * Indicates to be significant at a *P*-value less than (0.05) among Alg-nanoparticles with other groups. Furthermore, there were significant between control group (PBS) and other groups

**Figure 6:**
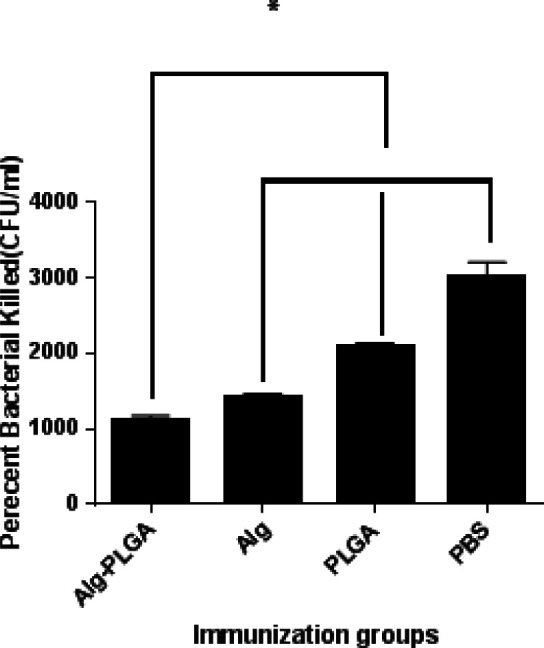
The opsonic killing activity of specific antisera versus *Pseudomonas aeruginosa *strain PAO1. Fourteen days after the third injection, all sera of experimental group were taken and pooled together

**Figure 7:**
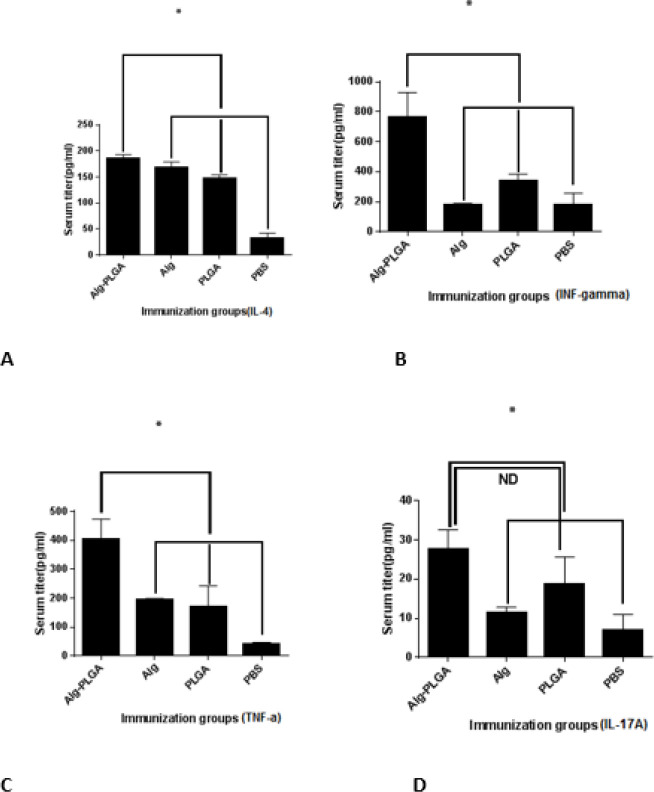
Evaluating cytokine responses in the immunized mice. The reported results are the mean of 3 mice in experimental group. Bars represent the means of triplicate determinations, and error bars indicate SD.) *) indicates significant differences (*P*<0.05) between Alginate-nanoparticles and other groups. (A) Alginate- PLGA, alginate, and PLGA had higher IL-4 and TNF-a cytokines, respectively. (B) Evaluating of INF- γ responses in the immunized mice. Alginate-PLGA had resulted in an INF-γ cytokine response. (C) Alginate- PLGA had higher TNF-α cytokine responses. (D) Alginate- PLGA group induced a significant IL-17A response, compared to other groups. Moreover, ND indicates no significant differences (*P*< 0.05) among Alg-nanoparticles and Alg in IL-17A responses

## Results

***Nanoparticle characterization and analysis***

Due to the results of size exclusion chromatography through Sephacryl S-200 HR, the tubes of alginate-PLGA (fraction numbers of 21 and 22), which contained high levels of nano-conjugates, was collected (Figure 1).

These tubes were selected, and the average hydrodynamic size of PLGA was measured via a Zetasizer instrument. Considering the results, the size and surface charge of nanoparticles (before binding to alginate) were 109 nm and -4.51 mv, respectively. After binding, the characterization of the charge in alginate-nanoparticles equaled 465.5 nm and -5.21 mv, respectively. The data predicated that binding were prosperity achieved.

FTIR test was done for the parts of PLGA. Due to FTIR data, the wave numbers from 1550 to 1810 cm^-1^ were the predicative of carboyl groups (at 1692.43 cm^-1^ and 1779.84 cm^-1^ for nanoparticles and alginate, respectively.

When the conjugation of alginate by PLGA nanoparticles was done, the alginate-PLGA samples’ peaks were observed at 1173.47 cm-1 and 1099.23 cm-1 for nanoparticles and alginate, respectively. These conversion in wave numbers were predictive of the formation of ester bonds in alginate - nanoparticles, verificating the event of the conjugation protocol (Figure 2). 

The three dimensional surface topography of alginate, PLGA, and alginate-PLGA nanoparticles were checked using Atomic Force Microscopy (AFM). Due to the concequence, the size of nanoparticles before alginate conjugated was in the range from 12 nm to 24 nm (Figure 3A and B). When the conjugation of nanoparticle was done, the size raised to 160-198 nm in the alginate-PLGA sample (Figure 3C and D). 

The shapes of surface binding grooves on nanoparticles were measured via AFM. After connection, the grooves were in the rounded form in alginate-PLGA, while before connection, these sites were sharp in PLGA. These data were indicative of the successful connection between alginate and PLGA.

***Antibody responses to immunization***

The anti-alginate, IgG was significantly raised in the mice which received alginate-PLGA, compared to all other groups of mice (Figure 4). These results indicated that alginate and alginate-PLGA are both suitable immunogens, though alginate-PLGA was more successful to motived the humoral immunity. No difference was significant between PLGA alone with control group (PBS) at (*P*<0.05). 

***Challenge test***

To peruse as the peculiar antibodies enhanced to the conjugated alginate-nanoparticles have efficacy to inhibit the distribution of *P. aeruginosa* into interior organs, we inquired the spread of infection via determining bacterial loads in the spleen. Then 72 hr of infection with PAO1 strain of *P. aeruginosa*, *P. aeruginosa* charge was measured in the spleens of immunized mice. 

Moreover, significant decreased bacterial titers were observed in the spleens the immunized mice infected by PAO1 strain, compared to PBS (control group) and PLGA groups (Figure 5). Furthermore, we establish that the antibodies enhanced to alginate-nanoparticles were more significantly effective to decrease the bacterial load, evaluated to the alginate group (*P*<0.05). Also we observed the mean difference is significant between control group and other groups (*P*<0.05). 

***Opsonic killing activity***

The opsonophagocytosis experiments were conducted to measure the functional activity of peculiar antibodies, which can intercede *P. aeruginosa* uptake by phagocytes, and their function is correlated to the clearance of infection. The antisera of IM immunized mice was taken and opsonic killing activity was determined *in vitro*.

 The antisera of immunized mice which had received alginate-PLGA were associated to significant killing levels of about 91% in a 1:4 dilution (Figure 6), suggesting that the candidate vaccine had induced a potent *P. aeruginosa* PAO1-specific antibody response. In mice, who had received control group, the phagocyte activity (2.3%) was apperceived. A significant difference was apperceived among the results of alginate-nanoparticles and PLGA groups (*P*<0.05). We also apperceived the average difference among (PBS) with other groups ( *P*<0.05). 

A remarkable opsonic killing activity was observed, when combined nanoparticle antiserum was treated to bacterial strain. Bars represent means of duplicate determinations, and error bars indicate SD. Results were accepted to be significant at (*P*<0.05). The * indicates to be significant at (*P*<0.05) among alginate-nanoparticles with eperimental groups. Moreover, there were significant at (*P*<0.05) among the (PBS) and other groups. 

***Cytokine responses to immunization***

The cytokine profiles in blood samples of mice in each immunized group were determined fourteen- hours after the last boost immunization. Cytokine levels in the isolated sera of each group were determined through ELISA. INF-γ mediates TH1-cells response, while IL-4 enhances TH2 response.

Due to the cytokine results for immunization with alginate, only IL-4 was significantly elevated (*P*<0.05), compared to control groups (PLGA and PBS), and alginate was not influential on TNF-a, IL-17A, and INF-γ cytokine responses. However, as shown in (Figures 7), alginate- PLGA was able to significantly increase the levels of TNF-a, IL-4, IL-17A, and INF-γ (*P*< 0.05) in the immunized groups, compared to the control (PLGA and PBS) and alginate groups. 

These results indicate that alginate conjugate in PLGA has improved and developed more pathways in cytokine response in the immunized mice.

## Discussion

*P. aeruginosa* is the bacteria that causes of life-treating infections in patients with Cystic Fibrosis (CF). These infections are because of alginate ability to create biofilms which display tolerance and resistance to antimicrobial agents. Because, the Alg plays a virulence role in adherence and colonization of this bacteria in respiratory epithelium which doesn’t toxic activity in cells (34-39). Therefore, the control and prevention of *P. aeruginosa* infection is a great concern. Hence alginate, which is a surface antigen, is a good vaccine candidate. Several studies showed that the protected epitope of alginate in PAO1 strains can be efficient to stimulate the immunity response and vaccine expansion in the patient with (CF) infection (35, 36, 39, 40, 44).

 Immunity against alginate can be efficient in eradicating the *P. aeruginosa *from patients with (CF). Introducing effective and safe in expensive carrier systems is the best issues in developing of vaccine candidate (45-49).

 We know the efficacy of a vaccine candidate strongly is related to select an appropriate carrier. PLGA are decomposable and polymeric matrices. As a result, vaccines composed of PLGA are a new part of vaccine which are more efficient and safe to the organs and more economical than coventional vaccine(52, 53, 56, 57-60). 

Therefore in the research, we built and distingushed novel nanoparticles-based vaccine candidates including alginate from *P. aeruginosa*. Moreover, we analyzed the candidate Alg-PLGA vaccine against this bacteria. Challenge test was assessed in the immunized mice, with the PAO1 strain of *P. aeruginosa,* and the functional activity of the conjugated PLGA was measured based on the *in vitro* opsonophagocytosis test, antibodies titer, and cytokine responses. 

Cytokines have various functions in hosts, depending on the bacterial antigen type and site.

The physiochemical studies showed that conjugation was successfully done. Furthermore, these candidate vaccines have indicated some advantages such as appropriate antigen delivery, non-toxicity and induction of strong immune responses using low antigen levels.

 Other applications of these vaccines include increasing the drug absorption and penetration, alginate presentation to B lymphocytes for stimulation of peculiar antibody responses, and phagocytosis by cytotoxic T lymphocytes. 

 The immunization study showed that the Alg-PLGA group introduces more immunogenicity than the Alg alone group. The monitoring of antibody responses and cytokines response indicated that immunogenicity considerably increases in alginate-nanoparticles conjugate compared to the pure alginate in the mice model. In the candidate vaccine, pathogenic *P. aeruginosa *stimulates TH2 cells.

 Then TH2 cells were produced IL-4,INF-ɣ, and IL-17A cytokines responses to clear *P. aeruginosa* strain PAO1, which mediated the recruitment and infiltration of polymorph nuclear leukocytes such as neutrophils. On the other hand, the production of TNF-a as an intermediate septic shock and INF-ɣ cytokines leading to activated macrophages, subsequently, opsonophagocytosis of *P. aeruginosa. *

The main finding of this research is that the candidate vaccine ( Alg-PLGA ) stimulated the motifs TLR and NodX with an unknown mechanism. Then, (NF-KB) transcription is activated and the cytokine genes such as TNF-a and INF-ɣ, with a synergistic mechanisms are expressed. 

INF-ɣ is stimulated innate immunity through macrophage receptors. These data suggested the macrophages, leading to the removal and clearance of *P. aeruginosa *in the host blood. The alginate-PLGA sample raised a broad immune response with a high antibody titer and activated cell-mediated immune responses through different pathways and elevation of the opsonic killing activity (compared to the alginate alone vaccine).

Also in this study, we observed a significant increase in the antibody titer and cytokines responses. 

## Conclusion

To concludes, the conjugation of *P.* *aeruginosa* alginate to the nanoparticle with Ethyl-3-(3-dimethylaminopropyl) carbodiimide as spacer molecules increment the functional activity of nano-vaccine through decreasing the bacterial propagation and increasing the killing of opsonized bacteria. This research was a basis for the subsequent expansion of a candidate vaccine for possible usage in humans to defend them against patient with this bacteria*.*
